# Orthostatic intolerance symptoms are associated with depression and diminished quality of life in patients with postural tachycardia syndrome

**DOI:** 10.1186/s12955-016-0548-x

**Published:** 2016-10-12

**Authors:** Jangsup Moon, Do-Yong Kim, Jung-Ick Byun, Jun-Sang Sunwoo, Jung-Ah Lim, Tae-Joon Kim, Jung-Won Shin, Woo-Jin Lee, Han Sang Lee, Jin-Sun Jun, Kyung-Il Park, Keun-Hwa Jung, Soon-Tae Lee, Ki-Young Jung, Kon Chu, Sang Kun Lee

**Affiliations:** 1 Department of Neurology, Laboratory for Neurotherapeutics, Comprehensive Epilepsy Center, Center for Medical Innovations, Biomedical Research Institute, Seoul National University Hospital, Seoul, Republic of Korea; 2 Department of Neurology, Seoul National University Hospital Healthcare System Gangnam Center, Seoul, Republic of Korea; 3 Program in Neuroscience, Seoul National University College of Medicine, Seoul, Republic of Korea; 4 Department of Neurology, Kyung Hee University Hospital at Gangdong, Seoul, Republic of Korea; 5 Department of Neurology, Soonchunhyang University Seoul Hospital, Seoul, Republic of Korea; 6 Department of Neurology, CHA Bundang Medical Center, CHA University, Seoungnam, Republic of Korea

**Keywords:** Orthostatic intolerance, Depression, Quality of life, Correlation, Postural tachycardia syndrome

## Abstract

**Background:**

Patients with postural tachycardia syndrome often appear depressive and report diminished quality of life (QOL). In the current study, we first evaluated if the maximal heart rate (HR) increment after standing is associated with the clinical symptoms in patients with excessive orthostatic tachycardia (OT). Next, we investigated the correlations among the symptoms of orthostatic intolerance (OI), depression, and health-related QOL in these patients. Finally we assessed if patients with minimal OI symptoms suffer from depression or diminished QOL.

**Methods:**

We performed a comprehensive questionnaire-based assessment of symptoms in 107 patients with excessive OT with a ≥ 30 beats/min heart rate increment (or ≥ 40 beats/min in individuals aged between 12 and 19) within 10 min after standing up. An existing orthostatic intolerance questionnaire (OIQ), the Beck depression inventory-II (BDI-II), and the 36 Item Short-Form Health Survey were completed prior to any treatment. Correlation analyses among the items of the questionnaires and other parameters were performed. Additionally, patients with minimal OI symptoms were analysed separately.

**Results:**

The maximal orthostatic HR increment was not associated with the clinical symptoms. The OI symptoms were significantly correlated with depression and diminished QOL. The BDI-II score demonstrated a positive linear relationship with total OIQ score (*r* = 0.516), and both physical and mental component summary scales of SF-36 showed a negative linear relationship with total OIQ score (*r* = -0.542 and *r* = -0.440, respectively; all *p* <0.001). Some OI symptoms were more strongly associated with depression, and others were more strongly related to QOL. Chest discomfort and concentration difficulties were the most influential OI symptoms for depression, while nausea and concentration difficulties were the most influential symptoms for physical and mental QOL, respectively. Dizziness and headache were the two most common complaints in patients with mild to moderate OI symptoms. In addition, subjects with minimal OI symptoms also had considerable deterioration in QOL.

**Conclusion:**

The OI symptoms, but not the maximal HR increment, are significantly correlated with depression and diminished QOL in patients with excessive OT. Therefore, pervasive history taking is important when encountering patients with excessive OT.

**Electronic supplementary material:**

The online version of this article (doi:10.1186/s12955-016-0548-x) contains supplementary material, which is available to authorized users.

## Background

Postural tachycardia syndrome (POTS) is a clinical syndrome usually characterized by an excessive increment of heart rate (HR) upon standing with frequent symptoms of orthostatic intolerance (OI). A diagnosis of POTS requires a HR increment ≥ 30 beats/min (bpm) (or ≥ 40 bpm in individuals aged between 12 and 19) within 10 min after standing from a recumbent position, in the absence of orthostatic hypotension [1]. However, some reports have shown that a certain portion of healthy individuals experience a HR increment of ≥ 30 bpm after standing (up to ~30 %), especially in the morning [2]. Therefore, symptoms of OI may be crucial for making a diagnosis of POTS.

Some studies define POTS as when patients have a > 6-month history of OI symptoms [2–4] while other studies require only a > 3-month history of symptoms [5–7], and some studies even do not have criteria for a minimal disease duration [8–11]. Both 3- and 6-month periods are fairly arbitrary criterion to indicate a chronic condition. Moreover, symptoms of POTS range from well-recognized orthostatic dizziness and palpitation, to less recognized symptoms, such as fatigue, gastrointestinal dysfunction, and increased sweating. [12, 13] These less recognized non-orthostatic symptoms could be unnoticeable without clinical suspicion.

The prevalence of POTS is estimated at 0.2 % in the general population [1]; however, the exact prevalence remains unknown. POTS is still considered to be an under-recognized disorder [12], and its clinical significance is increasing, as it has been reported to be frequently accompanied by depression [11, 14], sleep problems [15], chronic fatigue syndrome [16, 17], and diminished quality of life (QOL) [15, 18]. However, it is not known if depression or deterioration of QOL occurs frequently in patients with minimal OI symptoms despite excessive orthostatic tachycardia (OT).

In this study, we hypothesized that each specific symptom of POTS may have a different clinical significance for the assessment of POTS or comorbid conditions, and patients with minimal OI symptoms might have depression or deteriorated QOL. Therefore, we performed a comprehensive questionnaire-based assessment of OI symptoms, depression and QOL in patients with excessive OT, including those with minimal OI symptoms. First, we evaluated if the maximal HR increment is associated with the severity of symptoms. Second, we analysed the correlations among the symptoms of OI, depression, and the physical and mental components of QOL. Finally, we assessed if patients with minimal OI symptoms suffer from depression or deterioration of QOL.

## Methods

### Participants

Patient with excessive OT were recruited from either neurology outpatient clinics or the Center for Epilepsy and Autonomic Disorders of the Seoul National University Hospital. The main causes of admission were recurrent dizziness, headaches, loss of consciousness, convulsive movements, or other paroxysmal symptoms requiring the exclusion of syncope or seizure disorders for accurate diagnosis. Orthostatic vital sign (OVS) tests were performed twice in every patient except for one patient who had one OVS test and one head-up tilt test. The OVS test was performed as described before.[19] The patients were in a supine position for ≥ 10 min before the baseline blood pressure (BP) and HR were measured. After the baseline measurement, patients stood upright without support and remained still right beside the bed. The BP and HR were checked immediately and at 1, 3, 5, and 10 min after standing. Maximal HR increment observed in each patient was calculated from the two OVS tests.

Patients were included when at least one of the tests met the following criteria: (1) HR increment ≥ 30 bpm (or ≥ 40 bpm in patients aged between 12 and 19) within 10 min after standing up; (2) absence of orthostatic hypotension leading to compensatory orthostatic tachycardia; and (3) no overt cause for tachycardia, such as acute blood loss, hyperthyroidism, prolonged bed rest, or tachycardia-promoting medications. This study was approved by the Institutional Review Board of Seoul National University Hospital (IRB No. H-1401-091-550) and informed consent was obtained from all individual participants included in the study.

### Questionnaire

All subjects performed three sets of self-report questionnaires prior to the treatment. The symptoms of OI were evaluated using a questionnaire (Orthostatic Intolerance Questionnaire, OIQ) which has been widely used to assess the symptoms of POTS patients in previous studies [20–24]. The subjects specified the presence and frequency of nausea, tremor in hands, dizziness, palpitation, headache, profuse perspiration, blurred vision, chest discomfort, lightheadedness, and concentration difficulties. The frequency of specific symptoms was marked with a score ranging from 0 to 4, with 0 for never, 1 for once a month, 2 for 2–4 times per month, 3 for 2–7 times per week, and 4 for more often than once daily. The total symptom score was calculated by summing the scores of 10 symptoms.

Depression was assessed using the Beck depression inventory-II (BDI-II), which is a widely used self-report inventory with 21 multiple-choice questions (score of 0 to 3 for each question). A total score of 0–13 indicates minimal depression, 14–19 mild depression, 20–28 moderate depression, and 29–63 severe depression [25].

The 36 Item Short-Form Health Survey (SF-36) was used to assess the health related QOL [26]. The questionnaire contains 36 items that yield 8 category scales: physical functioning, role limitation caused by physical problems, bodily pain, general health, vitality, social functioning, role limitations caused by emotional problems, and mental health. These 8 category scores are aggregated into two summary scales, the physical component summary scale (PCS) and the mental component summary scale (MCS), which are normed to the population (mean = 50, standard deviation = 10). Higher score represents better QOL, and lower score indicates worse QOL.

### Statistics

Spearman’s rank correlation coefficient was performed to assess the correlation among variables. R-squared (r^2^) values were calculated to assess the goodness-of-fit for a linear model. To correct the potential confounding variables, partial correlation analysis was performed. To compare the mean among subgroups, a Kruskal-Wallis test was performed. The 95 % confidence interval (CI) was calculated from the sample mean and standard deviation [27]. Logistic regression analysis was performed to investigate the most influential OI symptom for depression or QOL. Significant depression was defined as BDI ≥ 14, and significant deterioration of physical or mental QOL were defined as PCS < 40 or MCS < 40, respectively. Univariate analysis for basic demographic factors (age, sex, body-mass index (BMI), maximal HR increment) and OIQ items were performed using *T*-test. The OIQ variables of *p* < 0.1 and/or basic demographic factors were included in the multivariate analysis. Data were collected and analysed using SPSS 22.0.0 for Windows, and values of *p* < 0.05 were considered significant.

## Results

### The maximal orthostatic heart rate increment is not associated with clinical symptoms

A total of 107 patients (67 females; mean age of 31.1 ± 1.3) were enrolled in this study. The mean BMI of the patients was 22.3 ± 0.3 (Table 1). In total, the mean value of maximal HR increment after standing up was 42.8 ± 1.2. The average of the total OIQ, BDI-II, PCS, and MCS scores were 14.9 ± 0.9, 14.8 ± 0.8, 43.4 ± 0.8, and 39.9 ± 1.1, respectively.

**Table 1:** Clinical characteristics of the patients

OIQ Total Score	Gender, F(%)	Age	Height (cm)	Weight (kg)	BMI	Max HR Increment	BDI-II	PCS	MCS
Total (*n* = 107)	67 (62.6%)	31.1 ± 1.3	166.3 ± 0.9	62.2 ± 1.3	22.3 ± 0.3	42.8 ± 1.2	14.8 ± 0.8	43.4 ± 0.8	39.9 ± 1.1
0–4 (*n* = 16)	7 (43.8%)	23.6 ± 3.0	169.2 ± 2.3	63.7 ± 2.8	22.2 ± 0.7	46.4 ± 3.0	7.4 ± 1.0	49.6 ± 2.2	46.9 ± 2.6
5–9 (*n* = 15)	9 (60%)	30.5 ± 2.4	168.8 ± 2.6	65.4 ± 3.9	22.7 ± 0.9	38.9 ± 3.0	10.2 ± 1.6	49.0 ± 1.7	45.4 ± 2.1
10–14 (*n* = 21)	13 (61.9%)	34.7 ± 4.0	168.5 ± 2.5	64.2 ± 3.6	22.4 ± 0.8	45.9 ± 2.9	13.3 ± 1.9	42.4 ± 1.3	41.0 ± 2.1
15–19 (*n* = 25)	16 (64%)	32.2 ± 2.3	165.2 ± 1.9	62.2 ± 3.1	22.6 ± 0.8	43.2 ± 2.8	17.2 ± 1.6	42.2 ± 1.3	38.8 ± 1.8
20–24 (*n* = 17)	14 (82.4%)	33.1 ± 2.3	161.5 ± 1.6	58.4 ± 2.4	22.4 ± 0.8	37.2 ± 1.5	19.1 ± 2.0	40.7 ± 1.5	35.9 ± 1.9
25–29 (*n* = 4)	4 (100%)	25.3 ± 3.2	160.1 ± 4.5	55.2 ± 4.9	21.4 ± 1.1	42.0 ± 4.6	21.8 ± 2.8	39.3 ± 3.7	29.7 ± 2.0
30–34 (*n* = 6)	3 (50%)	32.5 ± 6.3	167.8 ± 3.6	60.7 ± 4.3	21.5 ± 1.2	47.7 ± 3.9	21.5 ± 3.5	36.9 ± 2.2	30.1 ± 8.3
35–39 (*n* = 3)	1 (33.3%)	33.7 ± 8.8	164.9 ± 4.2	57.1 ± 5.4	20.9 ± 1.2	40.0 ± 6.2	21.0 ± 4.9	32.3 ± 6.7	33.5 ± 10.1
*P* value (Kruskall-Wallis)		0.047	0.211	0.81	0.984	0.1	<0.001*	<0.001*	<0.001*

The values are the mean ± SEM. The Kruskal-Wallis test was performed to compare the mean among the subgroups

*Abbreviations*: *OIQ* orthostatic intolerance questionnaire, *F* female, *BMI* body-mass index, *HR* heart rate, *BDI*-*II* Beck depression inventory-II, *PCS* physical component summary scale of Short Form 36, *MCS* mental component summary scale of Short Form 36

* *p* < 0.01

Spearman’s correlation coefficient revealed that the amount of the orthostatic HR increment was not associated with any of the OI symptoms, depression, or QOL. The maximal HR increment did not correlate with either the OIQ, BDI-II, PCS, or MCS scores (all *p* > 0.05; Table 2).

**Table 2:** Correlations among the questionnaire parameters

(*n* = 107)	OIQ Total	BDI-II	PCS	MCS
OIQ Total Score		.571**	−.534**	−.436**
1. Nausea		.401**	−.379**	−.209*
2. Tremor in hands		.325**	−.157	−.176
3. Dizziness		.272**	−.462**	−.307**
4. Palpitation		.426**	−.390**	−.283**
5. Headache		.224*	−.434**	−.262**
6. Profuse perspiration		.272**	−.213*	−.096
7. Blurred vision		.339**	−.249**	−.248*
8. Chest discomfort		.518**	−.348**	−.399**
9. Lightheadedness		.413**	−.511**	−.335**
10. Concentration difficulties		.532**	−.404**	−.466**
Max HR increase	−.081	−.032	.123	.083

Spearman’s correlation coefficient was performed for all patients (*n* = 107). The values represent the Spearman’s rho correlation coefficient

*Abbreviations*: *OIQ* orthostatic intolerance questionnaire, *BDI*-*II* Beck depression inventory-II, *PCS* physical component summary scale of Short Form 36, *MCS* mental component summary scale of Short Form 36

** *p* < 0.01, * *p* < 0.05, Bold: stronger correlation

### Orthostatic intolerance symptoms significantly correlate with depression and quality of life

**Fig. 1:**
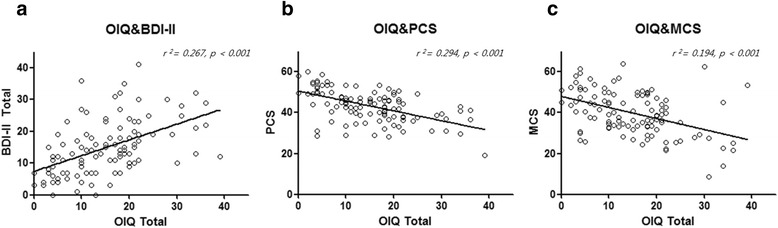
Correlation analyses between orthostatic intolerance symptoms and other questionnaire parameters. **a** The total orthostatic intolerance questionnaire (OIQ) score showed a significant positive linear correlation with the Beck depression inventory-II (BDI-II) score (*p* < 0.001). **b**, **c** The total OIQ score displayed a significant inverse linear correlation with the physical component summary scale (PCS) and the mental component summary scale (MCS) of the 36 Item Short-Form Health Survey (*p* < 0.001). *r* ^*2*^ represents a measure of goodness-of-fit of linear regression

Correlation analyses revealed that total OIQ score displayed a significant correlation with BDI-II, PCS, and MCS scores (all *p* < 0.001; Fig. 1). The BDI-II score demonstrated a positive linear relationship with total OIQ score, and both the PCS and MCS showed a negative linear relationship with total OIQ score. The strength of the correlation was highest in BDI-II (*r* = 0.571), followed by the PCS (*r* = -0.534) and MCS (*r* = -0.436; Table 2).

On partial correlation analysis, the BDI-II score displayed a positive linear correlation (*r* = 0.208, *p* = 0.029) with total OIQ score while controlling for PCS and MCS. PCS (*r* = -0.432, *p* < 0.01) and MCS (*r* = -0.198, *p* = 0.036) demonstrated inverse linear correlations with total OIQ score while controlling for BDI-II.

**Fig. 2:**
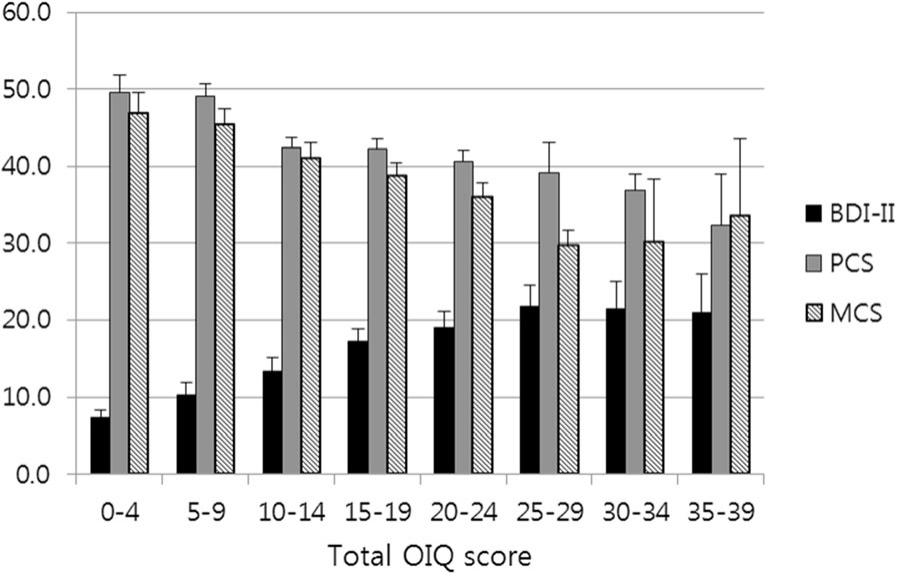
Mean values of BDI-II, PCS and MCS scores in subgroups categorized by total OIQ score. All patients were subcategorized according to total OIQ scores at intervals of 5 points. The mean BDI-II score in each group shows a tendency to increase as the OIQ score increases. The mean PCS and MCS values display a tendency to decrease as the total OIQ score decreases

When the patients were divided into 8 subgroups according to the OIQ scores, each group showed significantly different results on the BDI-II and SF-36 (*p* < 0.001; Table 1). There was a clear tendency that patients with a higher OIQ score had a greater BDI-II score and lower PCS and MCS scores (Fig. 2).

In summary, when the patients experienced more OI symptoms, they were more depressed and felt greater deterioration in their QOL.

### Particular symptoms may be more useful in screening patients with POTS

All contents of the OIQ were correlated with depression (BDI-II) and the physical (PCS) and mental components (MCS) of QOL, with the exception of a few items (Table 2). Tremor in hands did not correlate with PCS or MCS, and profuse perspiration did not correlate with MCS. Most of the 10 OI symptoms were significantly correlated with other symptoms except for a few combinations (Additional file 1: Table S1).

Assuming that the cases with a Spearman’s correlation coefficient *r* >0.4 indicate a stronger correlation, particular OI symptoms had a stronger association with BDI-II, PCS, and MCS scores (Table 2). Concentration difficulties, chest discomfort, palpitation, lightheadedness, and nausea were the symptoms most strongly associated with depression (in descending order). Lightheadedness, dizziness, headache, and concentration difficulties (in descending order) were strongly related to physical QOL, while concentration difficulties were strongly associated with mental QOL. On multivariate analysis (Table 3), patients with OIQ score ≥ 20 had significantly higher risk of having depression (odds ratio (OR) 14.300), diminished physical (OR 13.144) and mental QOL (OR 10.392) when compared to the patients with OIQ score <10. Among the OI symptoms, chest discomfort (OR 1.717) and concentration difficulties (OR 1.470) were the most influential symptoms for depression. Nausea (OR 2.501) and concentration difficulties (OR 1.756) were the most influential symptoms for physical and mental QOL, respectively (Table 3).

**Table 3:** Multivariate analysis for a significant functional deterioration

(*n* = 107)	Depression			Diminished physical QOL			Diminished mental QOL		
	OR	95 % CI	*P* value	OR	95 % CI	*P* value	OR	95 % CI	*P* value
Total OIQ score ^a^									
OIQ scores 0–9		Reference			Reference			Reference	
OIQ scores 10–19	6.190	2.021–18.958	0.001^*^	3.103	0.748–12.883	0.119	4.686	1.583–13.882	0.005^*^
OIQ scores ≥20	14.300	4.083–50.078	<0.001^*^	13.144	3.037–56.891	0.001^*^	10.392	3.071–35.163	<0.001^*^
OIQ score items ^b^									
Nausea				2.501	1.631–3.834	<0.001^*^			
Chest discomfort	1.717	1.130–2.610	0.011^*^						
Concentration difficulties	1.470	1.027–2.104	0.035^*^				1.756	1.290–2.389	<0.001^*^

*Abbreviations*: *OIQ* orthostatic intolerance questionnaire, *QOL* quality of life

^a^ Ordinal regression analysis was performed. Basic demographics (age, sex, body mass index, maximal HR increment) and total OIQ scores were analyzed

^b^ Logistic regression analysis was performed. Basic demographics (age, sex, body mass index, maximal HR increment) and OIQ items with *P* < 0.1 were analyzed using the backward Wald elimination method (cutoff *P* ≥ 0.05). The final OIQ items remained in the models are displayed

*p < 0.05

**Fig. 3:**
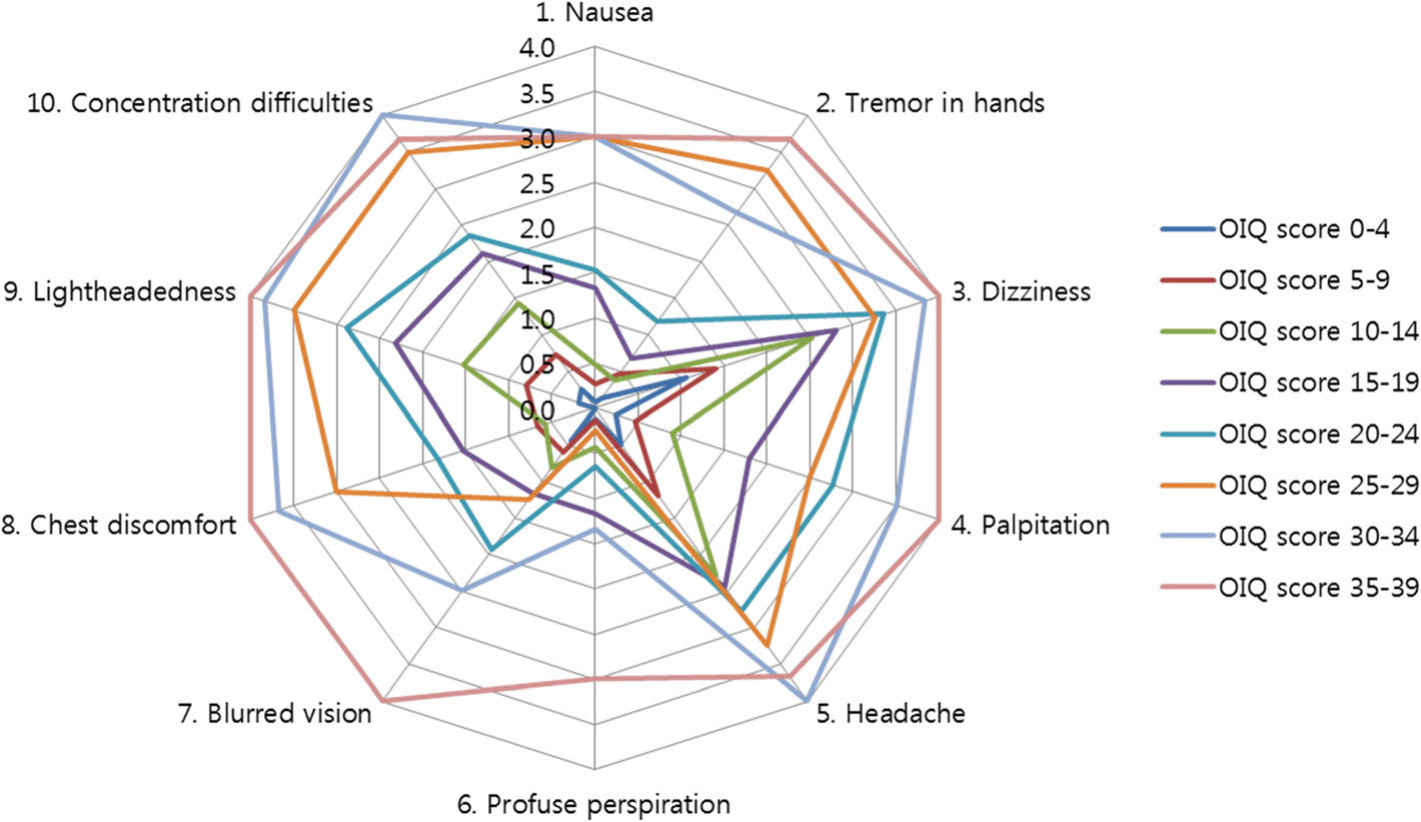
Symptoms self-reported by patients subcategorized according to total OIQ score. All patients were subcategorized according to total OIQ scores at intervals of 5 points. Each point of the decagonal scale represents the mean score of each symptom in the designated subgroup. For example, in the subgroup of patients with total OIQ score between 15 and 19 (*purple line*), the mean score of Nausea, Tremor in hands, Dizziness, and Palpitation was 1.3, 0.7, 2.8, and 1.8, respectively. Dizziness and headache were the two most common symptoms complained of by patients with an OIQ score < 20. As the OIQ score increased, patients tended to complain more about lightheadedness, concentration difficulties and palpitation. Profuse perspiration was the least reported patient complaint

When the patients were categorized into 8 subgroups according to the OIQ scores, certain symptoms appeared more frequently in patients with low total OIQ scores (Table 4, Fig. 3). Dizziness followed by headache were the two most common complaints in patients with an OIQ score <20. As the OIQ score increased, patients tended to complain more about lightheadedness, concentration difficulties and palpitation. In total, dizziness and headache were the most frequent symptoms, and profuse perspiration was the least reported patient complaint.

**Table 4:** Mean score of the OIQ items in each subgroup

(*n* = 107)	OIQ score								
	0–4 (*n* = 16)	5–9 (*n* = 15)	10–14 (*n* = 21)	15–19 (*n* = 25)	20–24 (*n* = 17)	25–29 (*n* = 4)	30–34 (*n* = 6)	35–39 (*n*- = 3)	Total
1. Nausea	0.1 ± 0.1	0.3 ± 0.2	0.5 ± 0.2	1.3 ± 0.3	1.5 ± 0.3	3.0 ± 0.7	3.0 ± 0.5	3.0 ± 0.6	1.1 ± 0.1
2. Tremor in hands	0.1 ± 0.1	0.5 ± 0.3	0.4 ± 0.2	0.7 ± 0.2	1.2 ± 0.2	3.3 ± 0.3	2.7 ± 0.3	3.7 ± 0.3	0.9 ± 0.1
3. Dizziness	**1.1 ± 0.3**	**1.4 ± 0.2**	**2.5 ± 0.3**	**2.8 ± 0.2**	**3.4 ± 0.2**	3.3 ± 0.5	3.8 ± 0.2	**4.0 ± 0.0**	**2.5 ± 0.1**
4. Palpitation	0.3 ± 0.1	0.5 ± 0.2	0.9 ± 0.2	1.8 ± 0.2	2.8 ± 0.3	2.5 ± 0.7	3.5 ± 0.2	**4.0 ± 0.0**	1.5 ± 0.1
5. Headache	**0.5 ± 0.2**	**1.2 ± 0.3**	**2.3 ± 0.3**	**2.4 ± 0.2**	2.8 ± 0.2	3.3 ± 0.5	**4.0 ± 0.0**	3.7 ± 0.3	**2.2 ± 0.1**
6. Profuse perspiration	0.1 ± 0.1	0.1 ± 0.1	0.4 ± 0.2	1.2 ± 0.2	0.6 ± 0.2	0.3 ± 0.3	1.3 ± 0.5	3.0 ± 0.6	0.7 ± 0.1
7. Blurred vision	0.4 ± 0.2	0.6 ± 0.2	0.8 ± 0.2	1.2 ± 0.2	1.9 ± 0.3	1.3 ± 0.8	2.5 ± 0.6	**4.0 ± 0.0**	1.2 ± 0.1
8. Chest discomfort	0.0 ± 0.0	0.7 ± 0.2	0.6 ± 0.2	1.5 ± 0.2	1.8 ± 0.3	3.0 ± 0.4	3.7 ± 0.2	**4.0 ± 0.0**	1.3 ± 0.1
9. Lightheadedness	0.2 ± 0.1	0.8 ± 0.2	1.5 ± 0.3	2.3 ± 0.2	**2.9 ± 0.2**	**3.5 ± 0.3**	3.8 ± 0.2	**4.0 ± 0.0**	1.9 ± 0.1
10. Concentration difficulties	0.3 ± 0.1	0.7 ± 0.2	1.4 ± 0.3	2.1 ± 0.2	2.4 ± 0.2	**3.5 ± 0.3**	**4.0 ± 0.0**	3.7 ± 0.3	1.7 ± 0.1
OIQ total	3.0 ± 0.3	6.7 ± 0.4	11.3 ± 0.3	17.3 ± 0.3	21.2 ± 0.3	26.8 ± 1.0	32.3 ± 0.8	37.0 ± 1.0	14.9 ± 0.9

The values are demonstrated as the mean ± SEM

The two most common symptoms in each group are displayed in bold characters

### Subjects with minimal orthostatic intolerance symptoms despite excessive orthostatic tachycardia may suffer from decreased quality of life

For the cut-off value distinguishing minimal and considerable OI symptoms, we chose a total OIQ score of 10. We regarded patients with OIQ score less than 10 as patients with minimal OI symptoms. In these subpopulations of patients with low OIQ scores between 0–4 and 5–9, MCS scores were substantially lower (46.9 ± 2.6 and 45.4 ± 2.1, respectively) than normal population, especially in patients with the OIQ score between 5–9 (MCS score 95 % CI: 41.0–49.8). However, PCS scores were close to the mean value of the healthy population (49.6 ± 2.2 and 49.0 ± 1.7, respectively) and BDI-II scores (7.4 ± 1.0 and 10.2 ± 1.6, respectively) were indicative of minimal depression (Table 1).

## Discussion

To our knowledge, this study is the first to evaluate associations between specific OI symptoms and depression or QOL in patients with POTS. The severity of each OI symptom was significantly associated with depression and QOL. The total OIQ score revealed a positive linear relationship with the BDI-II score and an inverse linear correlation with both PCS and MCS of the SF-36 scale. Meanwhile, the maximum HR increment after standing up was not associated with any of the OI symptoms, depression, or QOL. Certain OI symptoms were more strongly correlated with depression, and some were more strongly related to a diminished QOL. Dizziness and headache were the main complaints of patients with mild OI symptoms, and lightheadedness, concentration difficulties, and palpitation were more frequently observed in patients with moderate to severe OI symptoms. Patients with minimal OI symptoms also displayed deterioration of QOL.

Contrary to our expectation, the degree of orthostatic HR increment was not directly related to clinical symptoms, although excessive OT is the main clinical feature of POTS. This may have resulted from the fact that the time of the day when the OVS tests were performed varied among patients in our study. Diurnal variability of HR is considered a normal physiologic phenomenon [28, 29], and moreover it has been reported that OT is exaggerated in the morning, both in healthy control and in POTS patients [2, 19]. As the timing of the OVS test was not controlled, the orthostatic HR increment could have failed to reflect the severity of POTS. Alternatively, it is possible that the amount of the HR increment is truly not related to the severity of clinical symptoms. In any case, our finding implies that the severity of POTS should not be judged based on the amount of the HR increment after standing up.

Meanwhile, the severity of OI symptom was significantly correlated with depression and the QOL. As the OI symptoms increase, patients tend to be more depressed and feel deterioration in their QOL. The association between depression and decreased QOL is already well known in many diseases [30–32]. To exclude the possibility that depression and diminished QOL are the confounding variables, we performed a partial correlation analysis. The BDI-II score was significantly correlated with the OIQ score after correcting PCS and MCS, and likewise, PCS and MCS were also fairly related to the OIQ score after correcting for the BDI-II score. Moreover, on the multivariate analysis, patients with total OIQ score ≥ 20 had significantly higher risk of having depression and diminished physical and mental QOL (all OR > 10) than those with low OIQ scores. POTS patients are known to be mildly depressed, and their QOL is deteriorated [3, 15, 18]; however, here, we demonstrated that this deterioration is directly related to the OI symptoms. Although we cannot verify the causal relationship of OI symptoms and the psycho-behavioural symptoms, it is important to keep in mind that these symptoms are associated with each other. It is remarkable that patients with an OIQ score ≥ 10 demonstrated a mean BDI-II score over 13 and mean PCS and MCS scores below 42, which represent the presence of depression and significant impairment of QOL. We strongly recommend that in patients with excessive OT and an OIQ score ≥ 10, the clinician should evaluate the presence of coexisting depression or decline in QOL, which should be reassessed during the management of POTS patients. Antidepressants can be prescribed in severely depressed POTS patients and treatment of POTS may be continued in patients with sustained deterioration of QOL. We were not able to evaluate whether other comorbid conditions of POTS, such as sleep disturbance [15] or chronic fatigue syndrome [16], could be potential confounding factors; therefore, additional studies of this subject are required in the future.

We identified that some specific symptoms of OI are strongly correlated with depression and diminished QOL in POTS patients. Concentration difficulties, chest discomfort, palpitation, lightheadedness, and nausea showed the strongest correlations (in descending order) with depression while lightheadedness, dizziness, headache, and concentration difficulties had the strongest inverse correlations (in descending order) with the physical component of QOL. Concentration difficulties also displayed the strongest inverse correlation with the mental component of QOL. According to the multivariate analysis, chest discomfort and concentration difficulties were the most influential OI symptoms for depression, while nausea and concentration difficulties were the most influential symptoms for diminished physical and mental QOL, respectively. Benrud-Larson et al. previously demonstrated that autonomic symptoms are associated with QOL in POTS patients [18]. The orthostatic intolerance subscale showed the strongest correlation with PCS (*r* = -0.45, *p* < 0.05, Spearman’s correlation coefficient) but not with MCS of the SF-36 scale, and other subscales of autonomic symptoms displayed weaker correlations with QOL. The study used the Autonomic Symptom Profile [33] to evaluate the severity of autonomic symptoms. However, this measure consists of 169 questions, which require a substantial amount of time to complete the questions and to interpret the results. Therefore, in the current study, we focused on specific OI symptoms that can be easily asked to the patients in the clinic and thus have identified specific symptoms that are strongly associated with the PCS and MCS, as well as the BDI-II.

We discovered a pattern of frequently reported OI symptoms according to the total OIQ scores. Dizziness and headache were the most common symptoms complained of by patients with mild OI symptoms. Lightheadedness, concentration difficulties, and palpitations tended to increase in patients suffering from moderate to severe OI symptoms. We also discovered that profuse perspiration and blurred vision were not frequently presented in POTS patients with mild to moderate OI symptoms. Our data are additionally meaningful because we evaluated the baseline symptoms of OI, depression and QOL before the treatment was initiated. Most of the previous studies on depression and QOL in POTS patients assessed the patients after the treatment was begun [15, 18]. Therefore, we believe our data reflect the natural characteristics of POTS better than other previous studies. We estimate that the data on specific symptoms mentioned above will be highly useful in clinical practice dealing with POTS patients.

It is notable that, among the patients with excessive OT, even patients with minimal OI symptoms experienced deterioration in mental components of QOL. Although POTS patients often have a lower QOL [15, 18], it is not yet known whether the diminished QOL was the result of excessive OT or whether excessive OT occurs more frequently in patients with diminished QOL. Whether QOL improves after treatment of OT (e.g., beta-blockers) should be investigated in the near future to determine the causal relationship between the two conditions. At this point, we recommend assessing QOL and mood symptoms in patients who display excessive OT without significant OI symptoms, as they could be the underestimated symptoms of POTS.

## Conclusions

Our study confirms previous reports on the association between POTS and depression or diminished QOL. Furthermore, we identified that the severity of OI symptoms, but not the maximal HR increment, is significantly associated with depression and diminished QOL. Also, we revealed specific OI symptoms that have higher clinical significance in assessing psycho-behavioural aspects of POTS patients. We recommend clinicians to perform an organized and detailed history taking about the OI symptoms and psycho-behavioural symptoms (e.g., depression and decreased QOL) when assessing patients with excessive OT.

## Additional file

Additional file 1: Table S1. Correlations among the questionnaire parameters (full details). **Table S2.** Univariate analysis for a significant functional deterioration. (DOCX 34 kb)

## Abbreviations

BDI-II: Beck depression inventory-II
bpm: Beats/minute
CI: Confidence interval
HR: Heart rate
MCS: Mental component summary scale
OI: Orthostatic intolerance
OIQ: Orthostatic Intolerance Questionnaire
OT: Orthostatic tachycardia
OVS: Orthostatic vital sign
PCS: Physical component summary scale
POTS: Postural tachycardia syndrome
QOL: Quality of life
SF-36: 36 Item Short-Form Health Survey
