# Supplementary material for: Orthostatic intolerance symptoms are associated with depression and diminished quality of life in patients with postural tachycardia syndrome
Source: Health Qual Life Outcomes. 2016 Oct 12;14:144. doi: 10.1186/s12955-016-0548-x (PMC5059908; doi:10.1186/s12955-016-0548-x)
Supplement: Additional file 1: Table S1. — Correlations among the questionnaire parameters (full details). Table S2. Univariate analysis for a significant functional deterioration. (DOCX 34 kb) [file 12955_2016_548_MOESM1_ESM.docx]

**Table S1. Correlations among the questionnaire parameters (full details)**

| OIQ Items | OIQ Total | OIQ Items | | | | | | | | | | PCS | | | | MCS | | | |
| --- | --- | --- | --- | --- | --- | --- | --- | --- | --- | --- | --- | --- | --- | --- | --- | --- | --- | --- | --- |
|  |  | 1. | 2. | 3. | 4. | 5. | 6. | 7. | 8. | 9. | 10. | PF | RP | BP | GH | VT | SF | RE | MH |
| 1. Nausea | .676^**^ | 1 | .439^**^ | .390^**^ | .473^**^ | .385^**^ | .235^*^ | .246^*^ | .459^**^ | .414^**^ | .535^**^ | -.290^**^ | -.305^**^ | -.363^**^ | -.301^**^ | -.237^*^ | -.351^**^ | -.275^**^ | -.200^*^ |
| 2. Tremor in hands | .572^**^ | .439^**^ | 1 | .257^**^ | .453^**^ | .341^**^ | .220^*^ | .242^*^ | .424^**^ | .403^**^ | .366^**^ | -.133 | -.156 | -.222^*^ | -.171 | -.178 | -.234^*^ | -.150 | -.177 |
| 3. Dizziness | .676^**^ | .390^**^ | .257^**^ | 1 | .417^**^ | .537^**^ | .178 | .295^**^ | .363^**^ | .664^**^ | .294^**^ | -.331^**^ | -.391^**^ | -.490^**^ | -.387^**^ | -.383^**^ | -.402^**^ | -.312^**^ | -.299^**^ |
| 4. Palpitation | .728^**^ | .473^**^ | .453^**^ | .417^**^ | 1 | .381^**^ | .320^**^ | .275^**^ | .689^**^ | .410^**^ | .491^**^ | -.328^**^ | -.387^**^ | -.327^**^ | -.353^**^ | -.297^**^ | -.403^**^ | -.266^**^ | -.275^**^ |
| 5. Headache | .678^**^ | .385^**^ | .341^**^ | .537^**^ | .381^**^ | 1 | .287^**^ | .178 | .332^**^ | .624^**^ | .468^**^ | -.249^**^ | -.321^**^ | -.597^**^ | -.276^**^ | -.329^**^ | -.402^**^ | -.267^**^ | -.186 |
| 6. Profuse perspiration | .378^**^ | .235^*^ | .220^*^ | .178 | .320^**^ | .287^**^ | 1 | .157 | .186 | .260^**^ | .209^*^ | -.220^*^ | -.188 | -.193^*^ | -.168 | -.114 | -.195^*^ | -.174 | -.086 |
| 7. Blurred vision | .515^**^ | .246^*^ | .242^*^ | .295^**^ | .275^**^ | .178 | .157 | 1 | .426^**^ | .415^**^ | .277^**^ | -.277^**^ | -.184 | -.227^*^ | -.310^**^ | -.291^**^ | -.283^**^ | -.206^*^ | -.240^*^ |
| 8. Chest discomfort | .729^**^ | .459^**^ | .424^**^ | .363^**^ | .689^**^ | .332^**^ | .186 | .426^**^ | 1 | .468^**^ | .603^**^ | -.362^**^ | -.358^**^ | -.386^**^ | -.376^**^ | -.285^**^ | -.421^**^ | -.391^**^ | -.388^**^ |
| 9. Lightheadedness | .794^**^ | .414^**^ | .403^**^ | .664^**^ | .410^**^ | .624^**^ | .260^**^ | .415^**^ | .468^**^ | 1 | .498^**^ | -.392^**^ | -.349^**^ | -.547^**^ | -.405^**^ | -.386^**^ | -.481^**^ | -.321^**^ | -.299^**^ |
| 10. Concentration difficulties | .739^**^ | .535^**^ | .366^**^ | .294^**^ | .491^**^ | .468^**^ | .209^*^ | .277^**^ | .603^**^ | .498^**^ | 1 | -.360^**^ | -.406^**^ | -.442^**^ | -.374^**^ | -.405^**^ | -.476^**^ | -.434^**^ | -.465^**^ |

Spearman’s correlation analysis was performed for all patients (n=107). The values represent the Spearman’s rho correlation coefficient.
** p < 0.01, * p < 0.05, Bold: stronger correlation
Abbreviations: OIQ: orthostatic intolerance questionnaire, PCS: physical component summary scale of Short Form 36, MCS: mental component summary scale of Short Form 36, PF: physical functioning, RP: role limitation caused by physical problems, BP: bodily pain, GH: general health, VT: vitality, SF: social functioning, RE: role limitations caused by emotional problems, MH: mental health

**Table S2. Univariate analysis for a significant functional deterioration**

| **(n = 107)** | | **Depression** | | | **Diminished physical QOL** | | | **Diminished mental QOL** | | |  |
| --- | --- | --- | --- | --- | --- | --- | --- | --- | --- | --- | --- |
|  | | No  (n=55) | Yes (n=52) | *P* value | No  (n=72) | Yes  (n=35) | *P* value | No  (n=53) | Yes (n=54) | *P* value |  |
| Age, year | 30.0±13.9 | 32.2±12.1 | 0.398 | 27.9±9.7 | 37.7±16.3 | 0.182 | 28.0±11.6 | 34.1±13.7 | 0.014^*^ |  |  |
| Male Sex (%) | 25 (45.5) | 15 (28.8) | 0.076 | 30 (41.7) | 10 (28.6) | 0.002^*^ | 22 (41.5) | 18 (33.3) | 0.387 |  |  |
| Body Mass Index (kilogram/meter^2^) | 22.3±3.2 | 22.3±3.7 | 0.933 | 22.4±3.5 | 22.1±3.4 | 0.655 | 22.6±3.2 | 22.0±3.6 | 0.327 |  |  |
| Maximal HR Increment (beats/minute) | 42.8±11.3 | 42.7±12.9 | 0.970 | 43.1±11.7 | 42.2±12.9 | 0.732 | 43.4±11.9 | 42.2±12.2 | 0.589 |  |  |
| Total OIQ score | 11.3±8.4 | 18.7±7.9 | <0.001^*^ | 12.4±7.9 | 20.1±8.6 | <0.001^*^ | 11.7±8.5 | 18.0±8.2 | <0.001^*^ |  |  |
| **OIQ items** |  |  |  |  |  |  |  |  |  |  |  |
| 1. Nausea | 0.7±1.1 | 1.4±1.4 | 0.002^*^ | 0.7±1.0 | 1.8±1.5 | <0.001^*^ | 0.8±1.3 | 1.3±1.3 | 0.072 |  |  |
| 2. Tremor in hands | 0.6±1.1 | 1.2±1.3 | 0.024^*^ | 0.8±1.2 | 1.0±1.2 | 0.380 | 0.7±1.2 | 1.1±1.3 | 0.096 |  |  |
| 3. Dizziness | 2.3±1.4 | 2.7±1.2 | 0.060 | 2.2±1.3 | 3.1±1.2 | 0.001^*^ | 2.2±1.3 | 2.8±1.3 | 0.008^*^ |  |  |
| 4. Palpitation | 1.1±1.4 | 2.0±1.4 | 0.001^*^ | 1.3±1.3 | 2.1±1.5 | 0.002^*^ | 1.2±1.4 | 1.9±1.4 | 0.016^*^ |  |  |
| 5. Headache | 2.0±1.4 | 2.4±1.3 | 0.112 | 1.9±1.3 | 2.7±1.4 | 0.002^*^ | 1.8±1.4 | 2.5±1.2 | 0.007^*^ |  |  |
| 6. Profuse perspiration | 0.5±1.0 | 0.9±1.1 | 0.074 | 0.6±1.0 | 0.8±1.1 | 0.341 | 0.6±1.0 | 0.7±1.0 | 0.553 |  |  |
| 7. Blurred vision | 0.9±1.2 | 1.5±1.3 | 0.011^*^ | 1.0±1.2 | 1.6±1.4 | 0.025^*^ | 0.9±1.1 | 1.4±1.3 | 0.030^*^ |  |  |
| 8. Chest discomfort | 0.7±1.0 | 1.9±1.3 | <0.001^*^ | 1.0±1.2 | 1.8±1.4 | 0.007^*^ | 0.9±1.1 | 1.7±1.4 | 0.001^*^ |  |  |
| 9. Lightheadedness | 1.4±1.4 | 2.4±1.2 | <0.001^*^ | 1.5±.3 | 2.7±1.3 | <0.001^*^ | 1.5±1.5 | 2.3±1.3 | 0.002^*^ |  |  |
| 10. Concentration difficulties | 1.2±1.4 | 2.4±1.2 | <0.001^*^ | 1.4±1.4 | 2.5±1.4 | <0.001^*^ | 1.2±1.4 | 2.3±1.3 | <0.001^*^ |  |  |

The values are demonstrated as the mean ± standard deviation. T-test was performed to compare the mean.

Abbreviations: OIQ: orthostatic intolerance questionnaire, QOL: quality of life
